# Time-varying associations between diabetes and mortality following COVID-19: Evidence from a U.S. Veteran population

**DOI:** 10.1371/journal.pone.0333052

**Published:** 2025-10-08

**Authors:** Andrea R. Titus, Rania Kanchi, Samrachana Adhikari, Lorna E. Thorpe, David C. Lee, Aaron Baum, Mark D. Schwartz

**Affiliations:** 1 Department of Population Health, NYU Grossman School of Medicine, New York, New York, United States of America; 2 Ronald O. Perelman Department of Emergency Medicine, NYU Grossman School of Medicine, New York, New York, United States of America; 3 Department of Global Health, Icahn School of Medicine at Mount Sinai, New York, New York, United States of America; 4 VA New York Harbor Healthcare System, New York, New York, United States of America; The University of British Columbia, CANADA

## Abstract

Prior studies suggest that diabetes is associated with severe outcomes following COVID-19. However, most research has focused on early phases of the COVID-19 pandemic, and less is known about changing diabetes-associated risks over time. We constructed a retrospective cohort of U.S. Veterans with documented COVID-19 between March 2020 and August 2023 (N = 426,170). We used Poisson regression models to estimate relative risks of 60-day mortality following COVID-19 among Veterans with and without diabetes, incorporating demographic and clinical covariates, as well as weights to address unequal probabilities of selection into the sample. We then incorporated interaction terms representing six-month time windows and plotted predicted mortality risks over time. To contextualize risk estimates, we repeated the analysis among a cohort of Veterans without documented COVID-19. Diabetes was associated with overall higher risk of 60-day mortality following COVID-19 (RR = 1.21, 95% CI = 1.17–1.26). Mortality risks attenuated over time and converged with risks observed among Veterans without COVID-19 by March-August 2022. Results suggest that post-COVID-19 mortality risks associated with diabetes may have attenuated over time. Mechanisms underlying the attenuation of mortality risks were beyond the scope of the paper, however, future studies can potentially shed light on the contributions of population immunity (driven by previous infection or vaccination status), changing treatment patterns, and other factors to time-varying mortality risks following COVID-19 among individuals with diabetes.

## Introduction

Pre-existing type 2 diabetes has been shown to be associated with severe Coronavirus Disease 2019 (COVID-19) outcomes, including mortality [1–6]. A recent global umbrella meta-analysis estimated a pooled odds ratio of 1.87 (95% confidence interval (CI) = 1.61–2.17) for mortality following COVID-19 among individuals with diabetes, compared to those without [5]. Factors underlying associations between diabetes and mortality following COVID-19 are not fully established, but may include the contribution of diabetes to chronic inflammation and immune system dysfunction, as well as the presence of diabetes-associated comorbidities that may independently or synergistically contribute to worsened COVID-19 outcomes [7,8].

Most prior studies on the relationship between diabetes and COVID-19 outcomes focus on early waves of the pandemic (e.g., 2020 or 2021), and less is known about how the relationship between diabetes and COVID-19 severity has changed over time. Multiple factors may impact associations between risk factors (such as diabetes) and COVID-19 outcomes over time, including differences in SARS-CoV-2 (the pathogen that causes COVID-19) variants, the increasing prevalence of vaccination and prior infections that may alter immune response, and changes in treatment protocols [9,10]. In this retrospective cohort study, we leveraged over three years of electronic health record (EHR) data from the Veterans Health Administration to examine trends in mortality following documented COVID-19 among U.S. Veterans with and without diabetes.

## Methods

### Study population

Our sample was comprised of a subset of individuals included in the Veterans Administration Diabetes Risk (VADR) cohort [11]. Briefly, VADR is a dynamic cohort including adults (18+) with at least two outpatient visits within the Veterans Affairs (VA) health system more than 30 days apart between January 1, 2008 and January 1, 2019. Individuals were diabetes-free at cohort entry and were followed for subsequent diabetes incidence. For this analysis, we identified a sub-cohort of individuals who had at least one encounter at the VA health system in the two years prior to the start of the pandemic (March 1, 2020) and who had a VA-documented SARS-CoV-2 infection between March 1, 2020 and August 31, 2023. COVID-19 information was derived from the VA’s COVID-19 Shared Data Resource [12], which includes an indicator variable representing whether an individual was “ever positive” for COVID-19 based on documentation of a 1) positive VA PCR or antigen test or 2) evidence of COVID-19 in clinical notes (identified using natural language processing (NLP)). Each patient was assigned an index date based on their first positive PCR/antigen test or the inpatient admission date closest to the first positive test in the 15 days prior to the test. Individuals were excluded from the analytic sample if they had index dates outside of the study period or if they had missing covariate information. A flow chart showing sample exclusions at each stage is included in Supporting Fig 1. A graph with monthly counts of first documented COVID-19 within the analytic sample is included in S2 Fig.

**Fig 1:**
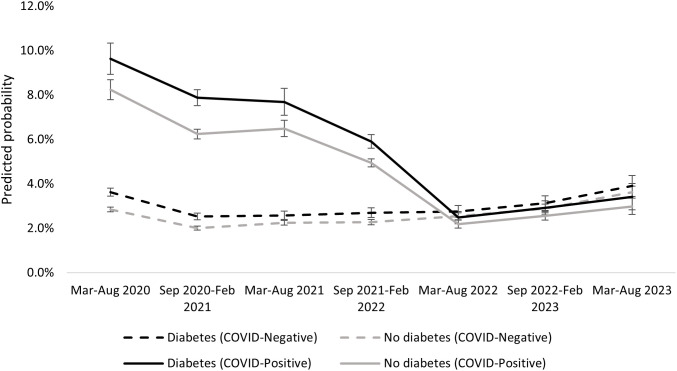
Average predicted probability of 60-day mortality following index date, by diabetes status and time (six-month windows). VADR cohort individuals with VA-documented COVID-19 (“COVID-Positive”) between March 1, 2020 and August 31, 2023 and VADR cohort individuals with VA-documented negative COVID-19 PCR test (“COVID-Negative”) between March 1, 2020 and August 31, 2023. Results generated from Poisson regression models that included interactions between diabetes status and time.

### Diabetes

Incident diabetes within the VADR cohort was defined as at least two encounters in the VA system with a type 2 diabetes-related International Classification of Disease (ICD)-9 or ICD-10 code, 2) documentation of a prescribed medication for diabetes (other than metformin or acarbose alone), or 3) at least one diabetes-related encounter along with two elevated HbA1C test results (defined as greater than or equal to 6.5%) [11]. Individuals were considered to have diabetes during the study period if they met the criteria for incident diabetes, ascertained through the beginning of the study period (March 1, 2020).

### Mortality

Death information was obtained from the VA COVID-19 Shared Data Resource. The outcome was defined as mortality (any cause) within 60 days of the index date. Our primary analysis focused on a 60-day post-infection window to ascertain mortality outcomes, in line with prior literature and with research suggesting that 60 days following a hospital admission for COVID-19 represents the highest risk period for mortality, particularly for individuals with severe symptoms [13]. The COVID-19 Shared Data Resource includes mortality information from three sources: the “SPatient” table within the VA’s Corporate Data Warehouse (CDW), the Master Veteran Index file, and the Vital Status Mini file. Information derived from these three sources has been used in prior studies and has been shown to be highly accurate in identifying mortality among U.S. Veterans [14–16].

### Covariates

Covariates were chosen based on hypothesized confounding pathways between our exposure (diabetes) and outcome (mortality following COVID-19) in a causal model. Regression models were adjusted for sex (male, female), race/ethnicity (non-Hispanic White, non-Hispanic Black, Hispanic, Non-Hispanic Asian, Non-Hispanic Native Hawaiian or Other Pacific Islander, Non-Hispanic American Indian or Alaskan Native, Unknown), and two variables representing age at index date: age category (less than 45, 45−59, 60−74, 75 or older) and continuous age. Race and ethnicity variables were constructed using self-reported information obtained from the CDW. We retained individuals with missing race and ethnicity information (5.6%) in an “Unknown” category. We also adjusted for the presence of several comorbid conditions at baseline (VADR cohort entry), including comorbidities that we hypothesized could be associated with incident diabetes and mortality, as well as comorbidities associated with mortality that may share common risk factors with incident diabetes. Comorbidities were measures using ICD-9 and ICD-10 diagnosis codes in inpatient and outpatient settings capturing history of cardiovascular and cerebrovascular disease (heart failure, ischemic heart disease, peripheral vascular disease, stroke), hypertension, chronic kidney disease, fatty liver disease, hepatitis C, depression or anxiety. We also adjusted for history of hyperlipidemia, defined as having at least two ICD codes for hyperlipidemia, a prescription for a lipid lowering medication, or total cholesterol greater than 240 mg/dL. All baseline variables were measured prior to diabetes incidence to avoid adjusting for factors that might mediate associations between diabetes and COVID-19 mortality. Other baseline covariates included smoking status (current smoker, former smoker, never smoker, unknown), body mass index (BMI), and a variable representing whether the individual was considered low-income or disabled (or neither), based on VA priority group [17]. The smoking status variable was derived from “Health Factor” data, based on a previously published algorithm [18]. Demographic variables, BMI, and priority group information were derived from the “SPatient” table and from tables capturing vital sign and enrollment information, available within the CDW.

### Statistical analysis

We examined descriptive statistics for the cohort as a whole and stratified by diabetes status. We estimated bivariate and adjusted Poisson regression models [19] to examine associations between diabetes and risk of mortality following COVID-19, incorporating the aforementioned covariates, as well as a categorical variable representing index date month. We then examined trends over time by including an interaction between diabetes and a categorical time variable representing seven 6-month pandemic windows between March 2020 and August 2023. The goal of this analysis was to examine whether associations between diabetes status and mortality following COVID-19 varied over time. We generated predicted mortality risks from these models and plotted probabilities of mortality for each time window (based on index date) for those with and without diabetes. To account for potential selection bias stemming from restricting to a study population with documentation of COVID-19 [20], all models included inverse probability of selection weights, incorporating the following variables: age (at cohort entry), age squared, sex, race/ethnicity, smoking status, BMI, disability/low-income status, and comorbidity history. Models to develop selection weights also included interactions between age and BMI, as well as between age and total number of comorbidities at baseline, as age, BMI, and comorbidities were hypothesized to be strong predictors of COVID-19 testing and mortality [20,21]. The propensity model to develop selection weights was estimated using logistic regression and included all individuals in VADR as of March 2020 who met inclusion criteria other than the criteria requiring documentation of COVID-19. Inclusion in the analytic sample was the dependent variable. Weighted outcome models were estimated with robust standard errors. Descriptive statistics for the analytic sample and for the overall VADR cohort are included in S1 Table. Differences between the two groups were relatively modest, though the analytic sample had higher proportions of females, younger individuals, and disabled individuals, compared to the VADR cohort. Veterans in the analytic sample were also more likely to be non-Hispanic Black and less likely to be non-Hispanic White.

Because it is plausible that individuals with diabetes have higher baseline mortality risks compared to individuals without diabetes, and that these baseline differences may drive associations observed in this study, we repeated our analysis within a secondary (“COVID-negative”) cohort of individuals, also derived from the COVID-19 Shared Data Resource. This cohort was comprised of Veterans with a documented negative SARS-CoV-2 PCR test and no evidence of COVID-19 during the study period (N = 885,973). Within this secondary cohort, we estimated associations between diabetes and 60-day mortality following an index date defined as each individual’s first negative PCR test (or hospitalization closest to the first negative PCR test within the 15 days prior to the test). We plotted predicted probabilities of mortality from this secondary analysis alongside results from the primary analysis.

As a sensitivity analysis, we re-estimated our primary model without selection weights. We estimated models with quarter-year time variables, rather than six-month time variables, and we plotted predicted probabilities from these models to examine whether time trends were similar with more granular time windows. We estimated models using an alternative outcome representing mortality within 30 days of the index date to examine whether we observed similar associations during a more acute phase of illness. We also conducted a sensitivity analysis using multiple imputation by chained equations to impute missing values for BMI, VA priority group, and smoking status [22]. Finally, because it is possible that differential time trends in mortality could be driven by more susceptible individuals experiencing mortality earlier on in the pandemic, we examined the characteristics of individuals who died following COVID-19 within each six-month time window, including mean number of baseline comorbidities, age, BMI, and disability/low-income status.

This study was reviewed and approved by the Subcommittee for Human Studies and the Research and Development Committee at the Department of Veterans Affairs. Data were accessed in January 2024 for research purposes. Investigators for this study had access to identifiable information to complete the analyses. Because this study includes sensitive health data and identifiable information, and data are not publicly available. Data can be accessed by VA employees or in collaboration with VA researchers [23]. Information regarding data access is available from the VA Information Research Center (VIReC) [24], and VIReC can be contacted via email at VIReC@va.gov. Analyses were conducted using Stata Statistical Software, version 18. Code for this analysis is available from the authors by request.

## Results

Descriptive statistics for the analytic sample are included in Table 1. The analytic cohort included 426,170 individuals, including 104,074 individuals with diabetes. Veterans with diabetes were more likely to be older and had higher mean BMI and comorbidity counts at VADR cohort entry. Bivariate and adjusted risk ratios (RRs) estimating associations between diabetes and mortality following COVID-19 are included in Table 2. Diabetes was associated with a higher risk of mortality following COVID-19 (adjusted RR = 1.21, 95% CI = 1.17–1.25). Regression results for all covariates from adjusted models are included in S2 Table.

**Table 1:** Descriptive statistics for the analytic sample, VADR cohort individuals with VA-documented COVID-19 between March 1, 2020-August 31, 2023.

	All	Diabetes^a^	No Diabetes
N	426,170	104,074	322,096
Sex (%)			
Male	87.8%	92.2%	86.3%
Female	12.3%	7.8%	13.7%
Age (%)			
Less than 45	18.4%	5.4%	22.6%
45-59	25.7%	23.6%	26.4%
60-74	40.7%	53.3%	36.6%
75+	15.2%	17.8%	14.4%
Mean age (SD)	60.3 (15.2)	65.2 (11.6)	58.7 (15.8)
Race/ethnicity (%)			
Non-Hispanic White	62.6%	60.2%	63.4%
Non-Hispanic Black	20.8%	23.7%	19.9%
Hispanic	8.6%	8.3%	8.7%
Non-Hispanic Asian	1.0%	0.8%	1.0%
Non-Hispanic Native Hawaiian/Pacific Islander	0.7%	0.8%	0.7%
Non-Hispanic American Indian/Alaskan Native	0.7%	0.7%	0.7%
Unknown	5.6%	5.4%	5.6%
Disability/low-income status (%)^b,c^			
Disabled	44.2%	43.3%	44.6%
Low-income	35.2%	37.0%	34.7%
Neither	20.5%	19.7%	20.8%
Smoking status (%)^c^			
Current smoker	26.2%	26.2%	26.2%
Former smoker	23.9%	26.9%	22.9%
Nonsmoker	33.5%	30.6%	34.5%
Unknown	16.4%	16.2%	16.4%
Mean number of comorbidities (SD)^c^	1.4 (1.3)	1.8 (1.4)	1.2 (1.2)
Mean BMI (SD)^c^	29.9 (5.5)	32.2 (6.0)	29.2 (5.2)

a)An individual was considered to have diabetes if they met the criteria for incident diabetes outlined in the manuscript.

b)Categorized based on VA priority group: “disabled” (priority groups 1–4); “low income” (priority groups 5 and 7); “neither” (priority groups 6 and 8).

c)Ascertained at VADR cohort entry, prior to documentation of diabetes diagnosis.

**Table 2:** Risk ratios (and 95% CIs) representing association between diabetes and 60-day mortality following COVID-19, VADR cohort individuals with VA-documented COVID-19 between March 1, 2020 and August 31, 2023 (N = 426,170).

	Bivariate RR (95% CI)	Adjusted RR (95% CI)^a^
Diabetes (no diabetes ref.)	1.41 (1.36-1.45)	1.21 (1.17-1.25)

a)Model adjusts for age category, continuous age, race/ethnicity, sex, index month, and the following variables ascertained at VADR cohort entry: smoking status, BMI, disability/low-income status, and comorbidity history (ischemic heart disease, heart failure, peripheral vascular disease, hypertension, stroke, chronic kidney disease, fatty liver disease, hepatitis C, hyperlipidemia, anxiety, and depression)

Fig 1 includes predicted probabilities of post-COVID-19 60-day mortality over time for the primary and “COVID-negative” cohorts, by diabetes status, generated from regression models including interactions between diabetes and six-month time windows. Mortality risks within the primary cohort were considerably higher than in the COVID-negative cohort early in the pandemic, though these risks converged by mid-2022. Between March and August 2020, the predicted probability of mortality following COVID-19 was 9.6% among those with diabetes, compared to 8.2% among those without diabetes (a risk difference of 1.4 percentage points). Corresponding probabilities were 3.4% and 3.0% between March and August 2023 (a risk difference of 0.4 percentage points). Within the COVID-negative cohort, mortality risks ranged from 2.5%−3.9% among individuals with diabetes and from 2.0%−3.6% among individuals without diabetes, and risk differences ranged from 0.2 to 0.8 percentage points. While absolute risks and risk differences attenuated over the study period in the primary cohort, risk ratios remained relatively constant.

RRs for models without selection weights were similar to the main analysis (S3 Table). RRs for models that incorporated imputation for missing data were also similar in direction and magnitude to the primary analysis (S4 Table). When using 30-day mortality as the outcome, instead of 60-day mortality, the adjusted RR was 1.20 (95% CI = 1.15–1.25) (S5 Table). Models incorporating quarter-year time windows (instead of six-month time windows) showed similar trends to the main analysis (S3 Fig). When examining the characteristics of those who experienced mortality following COVID-19, there were no clear time trends with regard to mean number of baseline comorbidities, age, or BMI (S6 Table). Individuals who died following COVID-19 in the first time window (March-August 2020) were more likely to be categorized as disabled (based on priority group), compared to later time windows. Mean age among those experiencing mortality also varied across the study period, with the lowest mean ages observed between March 2021 and February 2022.

## Discussion

Diabetes is a well-established risk factor for severe COVID-19 outcomes. However, most prior studies have focused on a relatively narrow time period early in the COVID-19 pandemic. In our analysis of over 400,000 U.S. Veterans with documented COVID-19 in the VA health care system between March 2020 and August 2023, diabetes was modestly associated with increased mortality. Our overall estimated risk ratio of 1.21, while smaller than some other published estimates [4–6], is in line with an estimated pooled mortality risk ratio associated with diabetes from U.S.-based studies of 1.15 (95% CI = 1.09–1.21) [6].

Our study encompassed over three years of data, which allowed us to examine post-COVID-19 mortality risks over time. Within our sample, risk ratios were relatively constant throughout the study period, though absolute mortality risks decreased over time among individuals with and without diabetes, and these risks converged with risks observed in our secondary, COVID-negative cohort in mid-2022. Mechanisms underlying the observed attenuation of mortality risks following COVID-19 were beyond the scope of this study but may include changes in individual immunity (e.g., due to vaccination or prior infection) [25], changes in clinical practices (including the introduction of pharmacotherapies) [10], or differences in the potential for severe outcomes associated with different SARS-CoV-2 variants [9]. For example, prior research within VA patient populations suggests that vaccine availability was associated with decreases in the proportion of COVID-19 patients with hypoxemia [26]. Case severity among Veterans has also been shown to be correlated with treatment, including receipt of antiviral medications [27]. Likewise, the characteristics and volume of individuals included in our analytic cohort over time may have been impacted by changes in COVID-19 testing access. For example, the substantial increase in at-home COVID-19 testing, beginning towards the end of 2021 [28], likely impacted the probability of healthcare-system based testing in later phases of the pandemic. Changes in the composition of patients included in the sample over time may contribute to time-varying mortality trends.

As a secondary analysis, we explored the possibility that individuals who were more susceptible to severe COVID-19 outcomes were more likely to die earlier in the pandemic [29]. We did not see strong evidence that individuals who died at earlier time points were different from those who died later in the study period based on observable characteristics, with the exception of disability status. However, individuals may have varied with regard to other health factors not measured in this analysis. It is important to note that our cohort represented individuals with relatively recent diabetes diagnoses (after January 2008), and it is plausible that mortality risks observed in this study may be more modest compared to studies of individuals with longer diabetes duration [30]. In addition, while we limited our analysis to examining mortality following first documentation of COVID-19, it is possible that individuals had other, preceding infections that were not captured within the VA EHR. The likelihood of misclassifying a “first” infection may have increased over time and may partially explain attenuated mortality risks if undocumented prior infections conferred protection against severe outcomes for individuals with index dates later in the study period [31].

Strengths of our analysis include the use of a “nested cohort” design with inverse probability weights to account for uneven probabilities of selection into the sample from the VADR study [20]. We leveraged a large dataset and over three years of data to examine time-varying associations. We adjusted for a range of baseline comorbidities captured in EHR data and we contextualized estimates by comparing to a secondary cohort of individuals with negative SARS-CoV-2 PCR tests.

Limitations included the potential for residual selection bias if all common causes of selection into the sample and the outcome were not included in the inverse probability weights or outcome regression models [20]. This residual selection bias could represent a “collider bias,” [20] however, even in the absence of collider bias, selection mechanisms can also lead to estimates that are biased with regard to the population of interest [32]. Our sample was derived from the VADR cohort, which represents an “in-care” patient population, as VADR eligibility criteria required at least two primary care visits to a VA facility within a five-year period. Restricting to an active VA patient population may help avoid misclassification stemming from challenges in distinguishing healthy individuals from those seeking health care outside the VA. However, this inclusion criteria may limit generalizability of results to the broader Veteran population. For example, prior research using data from the National Health Interview Survey suggests that Veterans who report seeking care at a VA facility in the past year have more health conditions and tend to have lower incomes, compared to Veterans as a whole [33]. We were not able to adjust for health conditions that were not captured or not measured in our data, and it is possible that our results were impacted by unmeasured confounding. However, we would hypothesize that residual confounding from other, unmeasured comorbid conditions would likely lead to an over-exaggeration of the association between diabetes and post-COVID-19 mortality, and we do not see strong evidence of this in time-varying models, given the convergence of estimates between our COVID-positive and COVID-negative cohorts. We included race and ethnicity as a covariate, however, race and ethnicity information captured within the EHR may be inaccurate or incomplete, and broad groupings may obscure important subgroup variation [34,35]. We could not ascertain if COVID-19 was the cause of mortality, as we did not have information on cause of death. We did not examine potential mediating factors driving higher mortality among those with diabetes, particularly early in the pandemic. Likewise, we did not examine whether time trends in post-COVID-19 mortality differed across subgroups of individuals with diabetes (e.g., stratified by glycemic control, diabetes duration, or medication use). This is an important avenue for future research.

Our secondary cohort of COVID-negative individuals was also likely not representative of the general VA population and included a substantial proportion of individuals with other morbidities (approximately 20% of this cohort was hospitalized on or within 60 days of their index date). In addition, it is possible that those who were identified as “COVID-negative” may have been misclassified and may have had undiagnosed or undocumented COVID-19. This misclassification may have been particularly pronounced in early phases of the pandemic and may help explain elevated risks of mortality within the COVID-negative cohort during the first time window (March-August 2020). Nevertheless, mortality risks derived from this secondary cohort can provide an alternative baseline to contextualize relative and absolute mortality risk differences associated with diabetes following COVID-19.

Within a cohort of U.S. Veterans, we found that diabetes was associated with an elevated mortality risk following COVID-19, however mortality risks among individuals with and without diabetes converged with risks among individuals without COVID-19 in later phases of the pandemic. Additional research can further elucidate mechanisms underlying COVID-19-associated mortality among individuals with underlying comorbidities, and can shed light on how the evolving landscape of SARS-CoV-2 variants, clinical practices, and individual and population-level immunity contribute to changing mortality patterns in high-risk populations.

## Supporting information

S1 Table Descriptive statistics for the analytic sample compared all potentially eligible individuals in VADR cohort. (DOCX)

S2 Table Beta coefficients (and 95% CIs) from primary regression model with 60-day mortality following COVID-19 as the outcome, VADR cohort individuals with VA-documented COVID-19 between March 1, 2020 and August 31, 2023 (N = 426,170). (DOCX)

S3 Table Risk ratios (and 95% CIs) representing association between diabetes history and 60-day mortality following COVID-19, VADR cohort individuals with VA-documented COVID-19 infection in VA between March 1, 2020 and August 31, 2023 (N = 426,170). Estimates from models that do not include selection weights.
(DOCX)

S4 Table Risk ratios (and 95% CIs) representing association between diabetes history and 60-day mortality following COVID-19, VADR cohort individuals with VA-documented COVID-19 infection in VA between March 1, 2020 and August 31, 2023 (N = 446,314). Estimates from models using multiple imputation to address missing data.
(DOCX)

S5 Table Risk ratios (and 95% CIs) representing association between diabetes history and 30-day mortality following COVID-19, VADR cohort individuals with VA-documented COVID-19 infection in VA between March 1, 2020 and August 31, 2023 (N = 426,170). (DOCX)

S6 Table Disability status, and mean number of comorbidities, BMI, and age among those who died following COVID-19 infection, by six-month pandemic windows. (DOCX)

S1 Fig Flow chart for primary analytic sample after applying exclusion criteria. (DOCX)

S2 Fig Monthly counts of first documented COVID-19, VADR cohort individuals with VA-documented COVID-19 between March 1, 2020 and August 31, 2023 (N = 426,170). (DOCX)

S3 Fig Average marginal effects representing predicted probability of 60-day mortality following index date, by diabetes status and time (three-month windows), VADR cohort individuals with VA-documented COVID-19 (“COVID-Positive”) between March 1, 2020 and August 31, 2023 and VADR cohort individuals with VA-documented negative COVID-19 PCR test (“COVID-Negative”) between March 1, 2020 and August 31, 2023. Results generated from Poisson regression models that included interactions between diabetes status and time.
(DOCX)
