# Supplementary material for: Time-varying associations between diabetes and mortality following COVID-19: Evidence from a U.S. Veteran population
Source: PLoS One. 2025 Oct 8;20(10):e0333052. doi: 10.1371/journal.pone.0333052 (PMC12507279; doi:10.1371/journal.pone.0333052)
Supplement: S1 Table — (DOCX) [file pone.0333052.s001.docx]

Supporting Table 1. Descriptive statistics for the analytic sample compared all potentially eligible individuals in VADR cohort

|  | Analytic sample | All VADR^a^ |
| --- | --- | --- |
| N | 426,170 | 3,618,917 |
|  |  |  |
| Sex (%) |  |  |
| Male | 87.8% | 90.9% |
| Female | 12.3% | 9.1% |
|  |  |  |
| Age (%)^b^ |  |  |
| Less than 45 | 31.5% | 24.4% |
| 45-59 | 34.6% | 30.4% |
| 60-74 | 29.5% | 35.8% |
| 75+ | 4.4% | 9.4% |
|  |  |  |
| Race/ethnicity (%) |  |  |
| Non-Hispanic White | 62.6% | 68.9% |
| Non-Hispanic Black | 20.8% | 16.7% |
| Hispanic | 8.6% | 6.0% |
| Non-Hispanic Asian | 1.0% | 0.9% |
| Non-Hispanic Native  Hawaiian/Pacific Islander | 0.7% | 0.7% |
| Non-Hispanic American  Indian/Alaskan Native | 0.7% | 0.6% |
| Unknown | 5.6% | 6.2% |
|  |  |  |
| Disability/low-income status (%) |  |  |
| Disabled | 44.2% | 40.6% |
| Low-income | 35.2% | 35.1% |
| Neither | 20.5% | 24.3% |
|  |  |  |
| Smoking status (%) |  |  |
| Current smoker | 26.2% | 28.3% |
| Former smoker | 23.9% | 24.6% |
| Never smoker | 33.5% | 30.6% |
| Unknown | 16.4% | 16.5% |
|  |  |  |
| Mean Number of comorbidities (SD) | 1.4 (1.3) | 1.4 (1.2) |
|  |  |  |
| Mean BMI (SD) | 29.9 (5.5) | 29.3 (5.3) |
|  |  |  |
| Mean age (SD)^b^ | 51.3 (14.4) | 55.1 (15.3) |

1. “All VADR” restricted to individuals who met similar baseline eligibility criteria (primary care visit to VA facility in 2 years prior to pandemic, non-missing covariate data), except for criteria requiring documentation of COVID-19 in the VA EHR
2. Represents age at cohort entry
