# Supplementary material for: Time-varying associations between diabetes and mortality following COVID-19: Evidence from a U.S. Veteran population
Source: PLoS One. 2025 Oct 8;20(10):e0333052. doi: 10.1371/journal.pone.0333052 (PMC12507279; doi:10.1371/journal.pone.0333052)
Supplement: S2 Table — (DOCX) [file pone.0333052.s002.docx]

Supporting Table 2. Beta coefficients (and 95% CIs) from primary regression model with 60-day mortality following COVID-19 as the outcome, VADR cohort individuals with VA-documented COVID-19 between March 1, 2020 and August 31, 2023 (N=426,170)^a^

| Variable | Beta coefficient (95% CI) |
| --- | --- |
| Diabetes (no diabetes ref.) | 1.21 (1.17-1.25) |
|  |  |
| Male (female ref.) | 1.72 (1.54-1.92) |
|  |  |
| Age category (<45 ref.) |  |
| 45-59 | 1.13 (0.96-1.34) |
| 60-74 | 1.59 (1.33-1.89) |
| 75+ | 1.76 (1.44-2.15) |
|  |  |
| Age (continuous) | 1.07 (1.07-1.08) |
|  |  |
| Race/ethnicity (non-Hispanic White ref.) |  |
| Non-Hispanic Black | 0.93 (0.89-0.97) |
| Hispanic | 1.00 (0.93-1.07) |
| Non-Hispanic Asian | 0.92 (0.72-1.16) |
| Non-Hispanic Native  Hawaiian/Pacific Islander | 1.15 (0.95-1.39) |
| Non-Hispanic American  Indian/Alaskan Native | 1.11 (0.91-1.35) |
| Unknown | 1.00 (0.93-1.07) |
|  |  |
| Disability/low-income status (Neither ref.)^b^ |  |
| Disabled | 1.11 (1.06-1.15) |
| Low-income | 1.24 (1.19-1.29) |
|  |  |
| Smoking status (Never smoker ref.) |  |
| Current smoker | 1.61 (1.54-1.68) |
| Former smoker | 1.15 (1.10-1.20) |
| Unknown | 1.12 (1.06-1.18) |
|  |  |
| Comorbidity history at baseline |  |
| Chronic kidney disease | 1.34 (1.24-1.45) |
| Fatty liver disease | 1.30 (1.05-1.62) |
| Heart failure | 1.22 (1.12-1.32) |
| Hepatitis C | 1.03 (0.92-1.15) |
| Hypertension | 1.11 (1.07-1.15) |
| Hyperlipidemia | 0.95 (0.91-0.98) |
| Ischemic heart disease | 1.09 (1.04-1.13) |
| Peripheral vascular disease | 1.22 (1.15-1.30) |
| Stroke | 1.64 (1.42-1.90) |
| Anxiety | 0.98 (0.93-1.02) |
| Depression | 1.05 (1.00-1.09) |
|  |  |
| Baseline BMI | 1.01 (1.00-1.01) |

1. Model also includes indicator variables for each month of the study period
2. Categorized based on VA priority group: “disabled” (priority groups 1-4); “low income” (priority groups 5 and 7); “neither” (priority groups 6 and 8)
