# Supplementary material for: Time-varying associations between diabetes and mortality following COVID-19: Evidence from a U.S. Veteran population
Source: PLoS One. 2025 Oct 8;20(10):e0333052. doi: 10.1371/journal.pone.0333052 (PMC12507279; doi:10.1371/journal.pone.0333052)
Supplement: S3 Table — Estimates from models that do not include selection weights. (DOCX) [file pone.0333052.s003.docx]

Supporting Table 3. Risk ratios (and 95% CIs) representing association between diabetes history and 60-day mortality following COVID-19, VADR cohort individuals with VA-documented COVID-19 infection in VA between March 1, 2020 and August 31, 2023 (N=426,170). Estimates from models that do not include selection weights.

|  | Bivariate RR (95% CI) | Adjusted RR (95% CI)^a^ |
| --- | --- | --- |
| Diabetes  (no diabetes ref.) | 1.67 (1.62-1.73) | 1.23 (1.19-1.28) |

1. Model adjusts for age category, continuous age, race/ethnicity, sex, index month, and the following variables ascertained at cohort entry: smoking status, BMI, disability/low-income status, and comorbidity history (ischemic heart disease, heart failure, peripheral vascular disease, hypertension, stroke, chronic kidney disease, fatty liver disease, hepatitis C, hyperlipidemia, anxiety, and depression)
