# Supplementary material for: Time-varying associations between diabetes and mortality following COVID-19: Evidence from a U.S. Veteran population
Source: PLoS One. 2025 Oct 8;20(10):e0333052. doi: 10.1371/journal.pone.0333052 (PMC12507279; doi:10.1371/journal.pone.0333052)
Supplement: S6 Table — (DOCX) [file pone.0333052.s006.docx]

Supporting Table 6. Disability status, and mean number of comorbidities, BMI, and age among those who died following COVID-19 infection, by six-month pandemic windows

| **Mar-Aug 2020** |  |
| --- | --- |
| N | 1877 |
| Disability/low-income status (%) |  |
| Disabled | 42.8% |
| Low-income | 39.9% |
| Neither | 17.3% |
| Mean number of comorbidities (SD) | 2.1 (1.5) |
| Mean BMI (SD) | 29.7 (5.7) |
| Mean age (SD) | 77.0 (11.3) |
|  |  |
| **Sep 2020-Feb 2021** |  |
| N | 4888 |
| Disability/low-income status (%) |  |
| Disabled | 34.6% |
| Low-income | 41.5% |
| Neither | 23.9% |
| Mean number of comorbidities (SD) | 2.0 (1.4) |
| Mean BMI (SD) | 29.9 (5.6) |
| Mean age (SD) | 76.9 (10.6) |
|  |  |
| **Mar-Aug 2021** |  |
| N | 1906 |
| Disability/low-income status (%) |  |
| Disabled | 36.7% |
| Low-income | 43.3% |
| Neither | 20.0% |
| Mean number of comorbidities (SD) | 1.8 (1.4) |
| Mean BMI (SD) | 30.2 (5.7) |
| Mean age (SD) | 71.4 (12.1) |
|  |  |
| **Sep 2021-Feb 2022** |  |
| N | 4406 |
| Disability/low-income status (%) |  |
| Disabled | 35.0% |
| Low-income | 43.3% |
| Neither | 21.8% |
| Mean number of comorbidities (SD) | 1.8 (1.5) |
| Mean BMI (SD) | 29.9 (5.8) |
| Mean age (SD) | 73.6 (11.3) |
|  |  |
| **Mar-Aug 2022** |  |
| N | 992 |
| Disability/low-income status (%) |  |
| Disabled | 31.7% |
| Low-income | 47.0% |
| Neither | 21.4% |
| Mean number of comorbidities (SD) | 1.9 (1.4) |
| Mean BMI (SD) | 28.6 (5.4) |
| Mean age (SD) | 76.6 (11.0) |
|  |  |
| **Sep 2022-Feb 2023** |  |
| N | 1057 |
| Disability/low-income status (%) |  |
| Disabled | 32.2% |
| Low-income | 47.5% |
| Neither | 20.3% |
| Mean number of comorbidities (SD) | 2.1 (1.5) |
| Mean BMI (SD) | 28.6 (5.7) |
| Mean age (SD) | 76.4 (10.5) |
|  |  |
| **Mar-Aug 2023** |  |
| N | 404 |
| Disability/low-income status (%) |  |
| Disabled | 34.2% |
| Low-income | 46.3% |
| Neither | 19.6% |
| Mean number of comorbidities (SD) | 1.9 (1.4) |
| Mean BMI (SD) | 28.7 (5.7) |
| Mean age (SD) | 76.7 (10.3) |
