# Supplementary figures and images for: Time-varying associations between diabetes and mortality following COVID-19: Evidence from a U.S. Veteran population
Source: PLoS One. 2025 Oct 8;20(10):e0333052. doi: 10.1371/journal.pone.0333052 (PMC12507279; doi:10.1371/journal.pone.0333052)

Supporting Figure 1. Flow chart for primary analytic sample after applying exclusion criteria


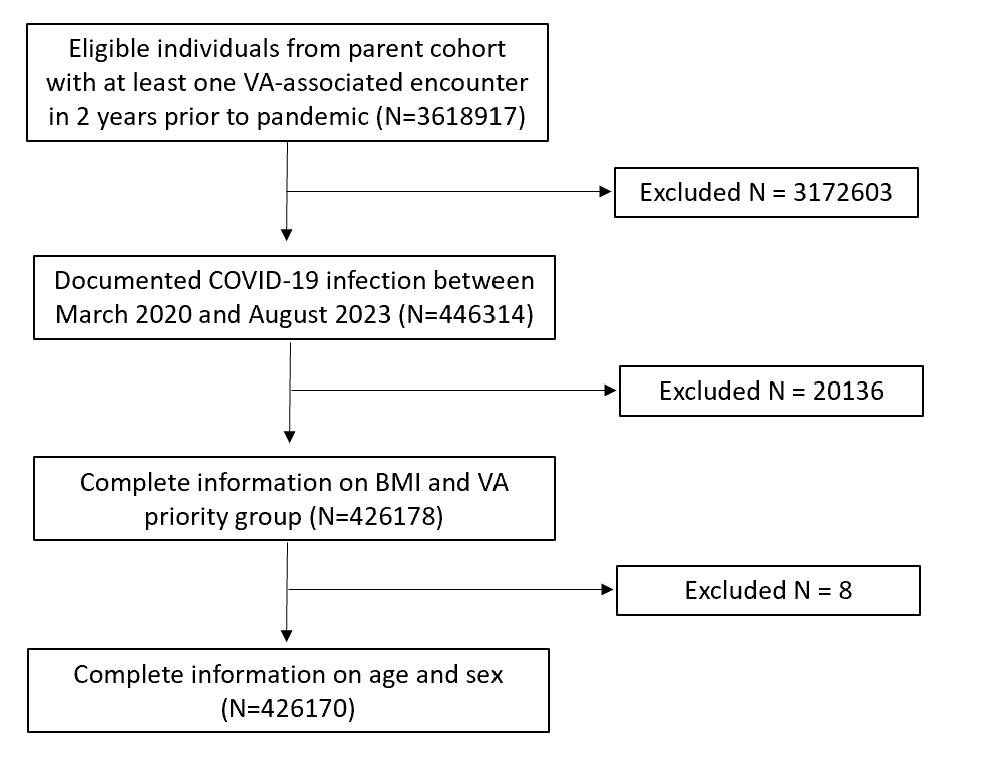

Supplement: S1 Fig — (DOCX) [file pone.0333052.s007.docx]
