# Supplementary material for: Time-varying associations between diabetes and mortality following COVID-19: Evidence from a U.S. Veteran population
Source: PLoS One. 2025 Oct 8;20(10):e0333052. doi: 10.1371/journal.pone.0333052 (PMC12507279; doi:10.1371/journal.pone.0333052)
Supplement: S2 Fig — (DOCX) [file pone.0333052.s008.docx]

Supporting Figure 2. Monthly counts of first documented COVID-19, VADR cohort individuals with VA-documented COVID-19 between March 1, 2020 and August 31, 2023 (N=426,170)
