# Supplementary material for: Time-varying associations between diabetes and mortality following COVID-19: Evidence from a U.S. Veteran population
Source: PLoS One. 2025 Oct 8;20(10):e0333052. doi: 10.1371/journal.pone.0333052 (PMC12507279; doi:10.1371/journal.pone.0333052)
Supplement: S3 Fig — Results generated from Poisson regression models that included interactions between diabetes status and time. (DOCX) [file pone.0333052.s009.docx]

Supporting Figure 3. Average marginal effects representing predicted probability of 60-day mortality following index date, by diabetes status and time (three-month windows), VADR cohort individuals with VA-documented COVID-19 (“COVID-Positive”) between March 1, 2020 and August 31, 2023 and VADR cohort individuals with VA-documented negative COVID-19 PCR test (“COVID-Negative”) between March 1, 2020 and August 31, 2023. Results generated from Poisson regression models that included interactions between diabetes status and time.
